# Isolationsmaßnahmen, Diagnostik und Organisation in deutschen Notaufnahmen während der COVID-19-Pandemie 2020

**DOI:** 10.1007/s00063-021-00775-7

**Published:** 2021-01-24

**Authors:** M. Finke, M. Pin, M. Bernhard, A. Rovas, H.-J. Pavenstädt, P. Kümpers

**Affiliations:** 1 Medizinische Klinik D (Allg. Innere Medizin und Notaufnahme sowie Nieren- und Hochdruckkrankheiten und Rheumatologie), Universitätsklinikum Münster, Albert-Schweitzer-Campus 1, Gebäude A1, 48149 Münster, Deutschland; 2 Zentrale interdisziplinäre Notaufnahme, Florence-Nightingale-Krankenhaus der Kaiserswerther Diakonie, Düsseldorf, Deutschland; 3 Zentrale Notaufnahme, Universitätsklinikum Düsseldorf, Düsseldorf, Deutschland

**Keywords:** Allokation, CO-RADS score, Algorithmus, Symptome, PCR Verfügbarkeit, Allocation, CO-RADS score, Algorithm, Symptoms, PCR availability

## Abstract

**Hintergrund:**

Die deutschen Notaufnahmen arbeiten seit Beginn der COVID-19-Pandemie im Spannungsfeld zwischen hoher Patientendichte und zusätzlichen anspruchsvollen hygienischen und organisatorischen Herausforderungen. Ziel dieser Studie war es einen Überblick über den aktuellen Stand bei Isolationsmaßnahmen, Diagnostik und Patientenallokation von COVID-19-Verdachtsfällen zu gewinnen.

**Methoden:**

Unterstützt durch die Deutsche Gesellschaft für Interdisziplinäre Notfall- und Akutmedizin (DGINA) befragten wir Notaufnahmeleiter*Innen im Rahmen einer anonymen Online-Umfrage zu Isolationsmaßnahmen, Diagnostik und Organisation in Notaufnahmen während der COVID-19-Pandemie.

**Ergebnisse:**

Insgesamt nahmen 139 Notaufnahmeleiter*Innen aus allen Bundesländern und allen Versorgungsstufen nach G-BA an der Umfrage teil. In fast allen teilnehmenden Notaufnahmen existieren schriftlich fixierte Verfahrensanweisungen zu COVID-19. Die meisten Notaufnahmen erfragen standardisiert die „klassischen“ COVID-19-Symptome wie Fieber, respiratorische Symptome oder Kontakt zu Corona-Patienten, wobei die Schwelle zur prophylaktischen Isolation sehr unterschiedlich hoch ist und konkrete Maßgaben zur Beendigung der Isolation häufig fehlen. Die individuellen Abstrich- und Allokations-Strategien variieren relativ stark. Weniger als die Hälfte aller Notaufnahmen haben ein gesondertes Procedere für nicht-anamnestizierbare Patienten (z.B. Polytrauma). In ca. 8 % der Verdachtsfälle erfolgt in der Notaufnahme eine COVID-19-spezifische thorakale Computertomographie.

**Schlussfolgerung:**

Zusammenfassend zeigt die aktuelle Umfrage, dass die deutschen Notaufnahmen für den Moment gut aufgestellt. Mit Blick auf eine mögliche Fallzahlsteigerung in den Wintermonaten wäre eine genauere Ausdifferenzierung der bisherigen Empfehlungen des Robert Koch Instituts, speziell für Notaufnahmepatienten, wünschenswert. In diesem Zusammenhang schlagen wir einen universalen Algorithmus zur (Ent-)Isolation von Verdachtsfällen in der Notaufnahme vor.

**Zusatzmaterial online:**

Die Onlineversion dieses Beitrags (10.1007/s00063-021-00775-7) enthält den Studienfragebogen. Beitrag und Zusatzmaterial stehen Ihnen auf www.springermedizin.de zur Verfügung. Bitte geben Sie dort den Beitragstitel in die Suche ein, das Zusatzmaterial finden Sie beim Beitrag unter „Ergänzende Inhalte“.

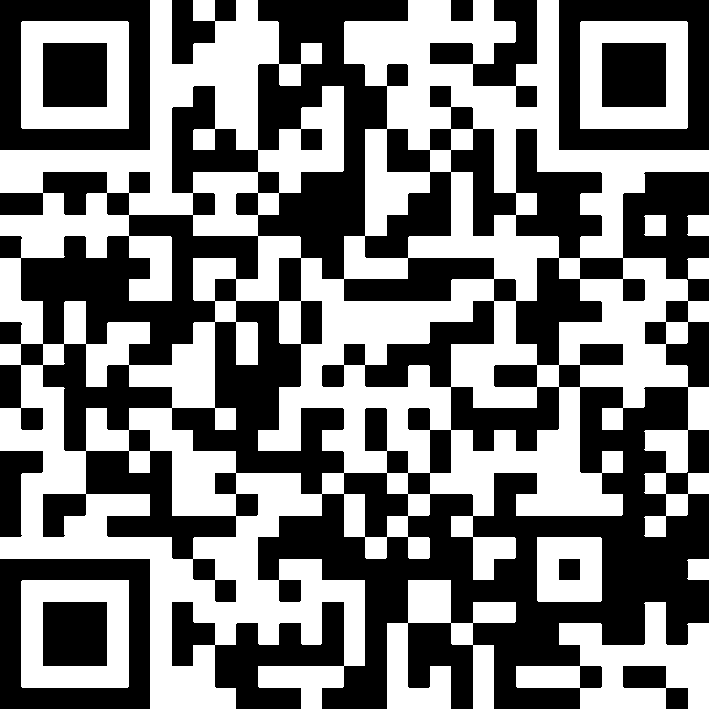

## Abstract (en)

**Background:**

Since the beginning of the coronavirus disease 19 (COVID-19) pandemic, German emergency departments (ED) have been working in the area of conflict between high case load and demanding hygienic and organizational challenges. The aim of this study was to gain an overview of the current status of isolation measures, diagnostics and patient allocation of suspected COVID-19 cases.

**Methods:**

Supported by the German Society for Interdisciplinary Emergency and Acute Medicine (DGINA) we invited leading ED physicians to answer an anonymous online survey regarding isolation measures, diagnostics and organization in emergency rooms during the COVID-19 pandemic.

**Results:**

A total of 139 responders from all federal states and all levels of care took part in the survey. Standard operating procedures on COVID-19 exist in almost all participating EDs, although concrete measures to end isolation are often missing. Most EDs screen patients for the “classic” COVID-19 symptoms such as fever, respiratory symptoms or contact to positive subjects in a standardized fashion, although the threshold for prophylactic isolation varies greatly. The individual swab-testing and allocation strategies vary relatively strongly. Less than half of all EDs have a separate procedure for uninterrogatable patients (e.g. major trauma). In about 8% of suspected cases, COVID-19-specific thoracic computed tomography is performed in the ED.

**Conclusion:**

The current survey shows that the German EDs are well positioned for the moment, even though the isolation threshold is too high at some locations. In view of a possible increase in the number of cases during the winter season, a more precise differentiation of the previous recommendations of the Robert Koch Institute, especially for emergency admission patients, would be desirable. In this context, we propose a universal algorithm for the (de-)isolation of suspect cases in the ED.

## Einleitung

In Zeiten der COVID-19-Pandemie stehen Notaufnahmen als primäre Anlaufstelle für den Rettungsdienst und fußläufige Patienten vor besonderen Herausforderungen [8, 13]. Im Gegensatz zur Intensivmedizin, wo der Fokus auf der Therapie dieser neuen Multisystemerkrankung liegt, stehen in der Notaufnahme die Identifikation, Risikostratifizierung und Allokation von Verdachtsfällen sowie die Organisation anspruchsvoller Hygienemaßnahmen eindeutig im Vordergrund.

In der Frühphase der Pandemie musste eine Vielzahl neuer Empfehlungen des Robert Koch-Instituts (RKI) zu Diagnostik, Infektionsschutzmaßnahmen, Prävention und Kontaktpersonenmanagement auf die jeweiligen lokalen Strukturen adaptiert und regelmäßig aktualisiert werden. Die entsprechenden Standard Operating Procedures (SOP), Flowcharts und Verfahrensanweisungen wurde in vielen Kliniken maßgeblich durch die Teams der Notaufnahmen entwickelt [8, 13].

Aktuell ist in Deutschland die sog. erste Welle der Pandemie überstanden und die Kliniken sind wieder weitestgehend zum Routinebetrieb übergegangen. Parallel dazu ändert sich in den Notaufnahmen die Perspektive: Statt der ursprünglich befürchteten Identifikation und umfassenden Erstversorgung von COVID-19-Fällen müssen nun relativ viele Akutpatienten einer aufwändigen COVID-19-Ausschlußdiagnostik unterzogen werden, um nosokomiale Transmissionen zu vermeiden und Patienten- und Mitarbeitersicherheit zu gewährleisten. Validierte Algorithmen stehen hierfür bislang nicht zur Verfügung.

Ziel dieser Studie war es daher, konkrete Maßnahmen und Strukturen, die in deutschen Notaufnahmen im Rahmen der COVID-19-Pandemie eingeführt oder geschaffen wurden, zu erfassen. Dabei wollten wir insbesondere einen Überblick über die Isolationsmaßnahmen, Diagnostik und Patientenallokation von Verdachtsfällen gewinnen.

## Methoden

Die Autoren entwickelten in einem Delphi-analogen Verfahren einen webbasierten Fragebogen, mit dem die organisatorischen Anpassungen an die Pandemiesituation erfragt wurden. Die Onlineumfrage umfasste insgesamt 42 Fragen aus den Bereichen: Screening/Diagnostik, Hygienemaßnahmen und persönliche Schutzausrüstung (PSA), Allokation von COVID-19-(Verdachts‑)Fällen, Personal und Reorganisation.

Alle Fragen wurden in deutscher Sprache gestellt und in der Umfragesoftware Typeform (https://www.typeform.com/) hinterlegt. Die Liste der Fragen ist im elektronischen Zusatzmaterial angehängt.

Eine Einladung zur Teilnahme wurde durch die Deutsche Gesellschaft für Interdisziplinäre Notfall- und Akutmedizin (DGINA) an Notaufnahmeleiter*Innen bzw. Ärzte*Innen in leitender Funktion in der Notaufnahme am 01.06.2020 versendet. Die Umfrage wurde am 24.06.2020 geschlossen.

Alle Antworten wurden auf Vollständigkeit geprüft und mittels SPSS Version 26.0.0.0 (IBM, Armonk, New York, USA) ausgewertet. Der Einfluss der verschiedenen Versorgungsstufen gemäß G‑BA wurde mittels χ^2^-Test (ordinale oder kategoriale Antworten) oder Kruskal-Wallis-Test mit Dunn-Test für Mehrfachvergleiche (metrische Daten) überprüft. Freitextantworten wurden separat gesammelt und individuell ausgewertet.

Da es sich um eine anonyme Befragung von Kollegen*Innen handelte und weder Patienten noch Patientendaten Bestandteil der Untersuchung waren, wurde kein Ethikvotum eingeholt.

## Ergebnisse

### Teilnehmer

**Figure 1:**
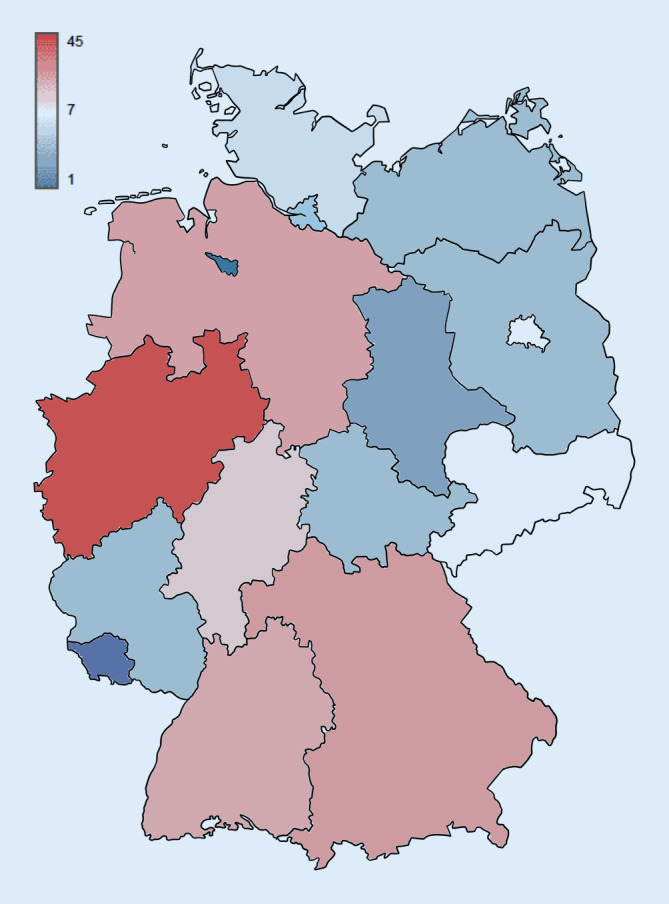

Die Rücklaufquote betrug 34,1 % (139/408). Insgesamt bearbeiteten 139 Notaufnahmeleiter*Innen bzw. Ärzte*Innen in leitender Funktion den Fragebogen vollständig. Von diesen arbeiteten 29,5 % (*n* = 41) in Notaufnahmen der Basisnotfallversorgung, 36,0 % (*n* = 50) in Notaufnahmen der erweiterten Notfallversorgung und 34,5 % (*n* = 48) in Notaufnahmen der umfassenden Notfallversorgung. Es waren dabei alle 16 deutschen Bundesländer vertreten, wobei Notaufnahmen aus dem bevölkerungsreichsten Bundesland Nordrhein-Westfalen den größten Anteil stellten (29,5 %; Abb. 1). Die Bettenzahl der untersuchten Krankenhäuser reichte von 100–1991 Betten, wobei die mediane Bettenzahl 500 (Interquartilbereich: 310–670) betrug.

### Verfahrensanweisungen und SOP

**Figure 2:**
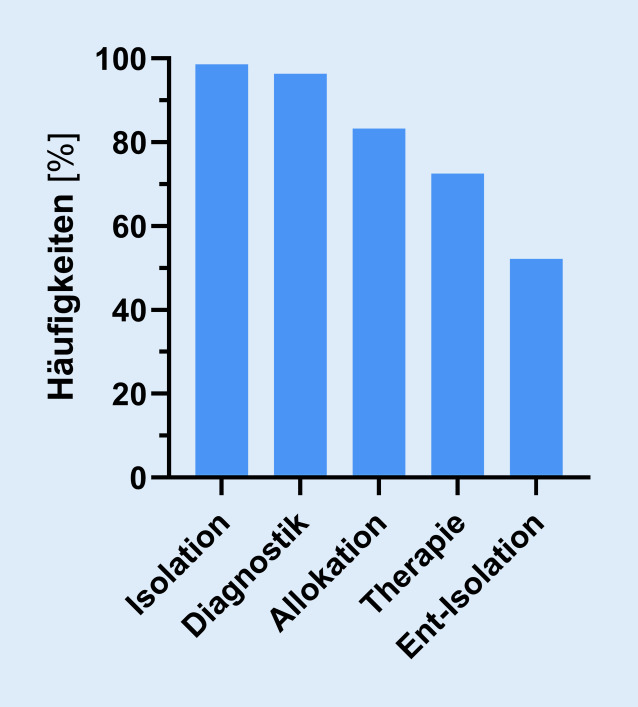

In 99,3 % (*n* = 138) der teilnehmenden Notaufnahmen existieren schriftlich fixierte Verfahrensanweisungen oder SOP zu COVID-19. Anweisungen zum Thema Isolation liegen in 98,6 % (*n* = 136), zum Thema Diagnostik in 96,4 % (*n* = 133), zum Thema Allokation/Verlegung in 83,3 % (*n* = 115) und zur Therapie in 72,5 % (*n* = 100) der Notaufnahmen vor. Schriftliche Anweisungen zur Entisolation von Verdachtspatienten existieren hingegen nur in 52,2 % (*n* = 72) der Notaufnahmen (Abb. 2).

### Screening

In 88,5 % (*n* = 123) der teilnehmenden Notaufnahmen werden Patienten mittels Fragebogen oder standardisiert-strukturierter Anamnese auf COVID-19-Symptome gescreent. Die häufigsten dabei abgefragten Symptome sind Fieber (100 %, *n* = 123), Husten (97,6 %, *n* = 120), Geschmacks- und/oder Geruchsverlust (95,9 %, *n* = 118) und Dyspnoe (90,2 %, *n* = 111). Andere Symptome werden deutlich seltener erfragt (Tab. 1). Im Rahmen dieses Screenings werden in 84,6 % der Notaufnahmen Vitalparameter erhoben. Alle Notaufnahmen messen dabei die Körpertemperatur, andere Vitalparameter werden mit unterschiedlicher Häufigkeit erhoben (Tab. 2). In nur 59,4 % der Notaufnahmen gibt es Festlegungen, wie bei nichtbefragbaren Patienten (Intoxikation, intubierter Patient, hochgradige Demenz etc.) vorzugehen ist.

**Table 1:** 

Symptom/Umstand	Wird erfragt in …	
	Prozent (%)	*n*
Fieber	100,0	123
Husten	97,6	120
Geschmacks- und/oder Geruchsverlust	95,9	118
Kontakt zu SARS-CoV-2-positiven Menschen	94,3	116
Dyspnoe	90,2	111
Halsschmerzen	76,4	94
Aufenthalt in einem Coronahotspot	65,0	80
Gliederschmerzen	58,5	72
Magen-Darm-Symptome	58,5	72
Abgeschlagenheit/Leistungsverlust	57,7	71
Schnupfen	56,1	69
Schluckstörungen	30,1	37
Aufenthalt in Krankenhaus/Pflegeheim	2,4	2

Notaufnahmen, die einen spezifischen Coronafragebogen oder eine standardisiert-strukturierte Anamnese zum Thema COVID-19 erheben (%, *n* = 123)

**Table 2:** 

Vitalparameter	Wird erhoben in …	
	Prozent (%)	*n*
Temperatur	100,0	104
Sauerstoffsättigung	93,3	97
Herzfrequenz	84,6	88
Atemfrequenz	81,1	85
Blutdruck	72,1	75
Vigilanz	66,3	69
Rekapillarisierungszeit	1,0	1

Notaufnahmen, die im Rahmen eines spezifischen Coronafragebogens oder einer standardisiert-strukturierten Anamnese Vitalparameter erheben (%, *n* = 104)

### Isolation

Wenn ein Coronafragebogen oder eine standardisiert-strukturierte Anamnese erhoben wird, so führt in 74,8 % der Notaufnahmen (*n* = 92) bereits *ein* positiv erfragtes Symptom zu einer unmittelbaren prophylaktischen Isolation der Patienten in der Notaufnahme. In 8,9 % (*n* = 11) der Notaufnahmen führen erst 2, in 1,6 % (*n* = 2) erst 3 oder mehr positiv erfragte Symptome zu einer prophylaktischen Isolation. Trotz standardisierter Screeningfragen bleibt in 13,8 % der Notaufnahmen (*n* = 17) die Isolation immer eine Einzelfallentscheidung des behandelnden Personals. Notaufnahmen, die keinen Coronafragebogen oder standardisiert-strukturierte Anamnese erheben (11,5 %, *n* = 16), isolieren meist aufgrund von Fieber, respiratorischen Symptomen oder positiver Kontaktanamnese.

Durchschnittlich werden 26 % aller Notaufnahmepatienten in den teilnehmenden Notaufnahmen abgestrichen und prophylaktisch isoliert (Median [IQR]: 15 [6,5–35] %). Die Häufigkeit der verwendeten Materialien zur prophylaktischen Isolation ist Tab. 3 zu entnehmen.

**Table 3:** 

Materialien	Wird zur prophylaktischen Isolation genutzt in …	
	Prozent (%)	*n*
Handschuhe	97,8	136
FFP2-Masken	95,0	132
Reguläre Schutzkittel	79,9	111
Schutzbrillen	79,1	110
Gesichtsschutz/„face shield“	68,3	95
„Chirurgische“ Schutzkittel	28,8	40
Chirurgische Masken	12,9	18
FFP3-Masken	11,5	16

### Abstriche/PCR-Diagnostik

Insgesamt 81,3 % der Befragten (*n* = 113) gaben an, dass Abstriche auf SARS-CoV‑2 in ihrer Notaufnahme überwiegend von Pflegepersonal durchgeführt würden. In 12,2 % bzw. 6,5 % der Notaufnahmen werden die Abstriche vom ärztlichen Personal bzw. medizinischen Fachangestellten durchgeführt. In 18 % der Notaufnahmen (*n* = 25) ist bereits ein Schnelltest auf SARS-CoV‑2 verfügbar. In den übrigen Notaufnahmen schätzen die Teilnehmer die Dauer bis zum Erhalt des Ergebnisses auf durchschnittlich 16 ± 10 h. Im Dienst bzw. am Wochenende verlängert sich diese Dauer auf durchschnittlich 18 ± 10 bzw. 23 ± 14 h.

Insgesamt 55,4 % (*n* = 77) der Teilnehmer gaben an, dass für ambulante SARS-CoV2-Testungen ein öffentliches Abstrichzentrum außerhalb des Krankenhauses genutzt wird. In 32,4 % der Fälle (*n* = 45) werden Abstriche außerhalb der Räumlichkeiten der Notaufnahme, aber innerhalb des Krankenhausgeländes durchgeführt (meist Containerlösungen). 12,2 % der Notaufnahmen (*n* = 17) führen diese Testung innerhalb der eigenen Räumlichkeiten durch.

Nur 5 % der teilnehmenden Notaufnahmen (*n* = 7) führen bei ausnahmslos allen Notfallpatienten Abstriche zwecks SARS-CoV2-Diagnostik durch. In 46 % (*n* = 64) der Notaufnahmen werden alle Patienten abgestrichen, bei denen die Indikation zur stationären Aufnahme besteht. Dem gegenüberstehend werden in 3,6 % der Notaufnahmen (*n* = 5) Patienten erst dann abgestrichen, wenn alternative Diagnosen ausgeschlossen wurden.

Patienten, die sonst im „Driveby-“ bzw. „Bypassverfahren“ an der Notaufnahme verbeigeführt wurden (z. B. STEMI direkt zum Herzkatheterlabor), werden nun in 35,5 % der Notaufnahmen (*n* = 49) zum Abstrich durch die Notaufnahmen umgeleitet.

Konstellationen, in denen ein zweites (negatives) PCR-Testergebnis zur Entisolation vorgeschrieben ist, werden von 21,2 % der Teilnehmer (*n* = 29) benannt. Eine Notwendigkeit hierzu wird vor allem bei hohem klinischem und/oder radiologischem Verdacht auf COVID-19 sowie einer geplanten (Rück‑)Verlegung gesehen.

In 15,1 % der Notaufnahmen (*n* = 21) werden die Mitarbeiter im Sinne einer Personalsurveillance regelmäßig abgestrichen. Wenn diese Abstriche durchgeführt werden, so liegen im Median 7 (Interquartilbereich: 7–14) Tage zwischen den Testungen.

#### Bildgebung

Die thorakale Computertomographie (CT) wird in 64,7 % der Notaufnahmen (*n* = 90) regelhaft zur (Ausschluss‑)Diagnostik bei COVID-19-Verdachtsfällen eingesetzt. In diesen Notaufnahmen wird eine CT im Median bei 29 % (Interquartilbereich: 5–40 %) aller COVID-19-(Verdachts‑)Fälle in Ergänzung zum Abstrich eingesetzt. Eine standardisierte, COVID-spezifische Befundung der CT erfolgt dann in 74,4 % der Kliniken. Hier genutzte Klassifikationssysteme sind vor allem die CORADS-Klassifikation [7], ein „chest CT score“ [5] sowie die „Klinische Anwendung und Aussagekraft der CT bei COVID-19 Epidemie“ der Deutschen Röntgengesellschaft [9]. Die Lungensonographie wird in lediglich 27,3 % der Notaufnahmen (*n* = 38) regelhaft eingesetzt.

### Allokation

**Figure 3:**
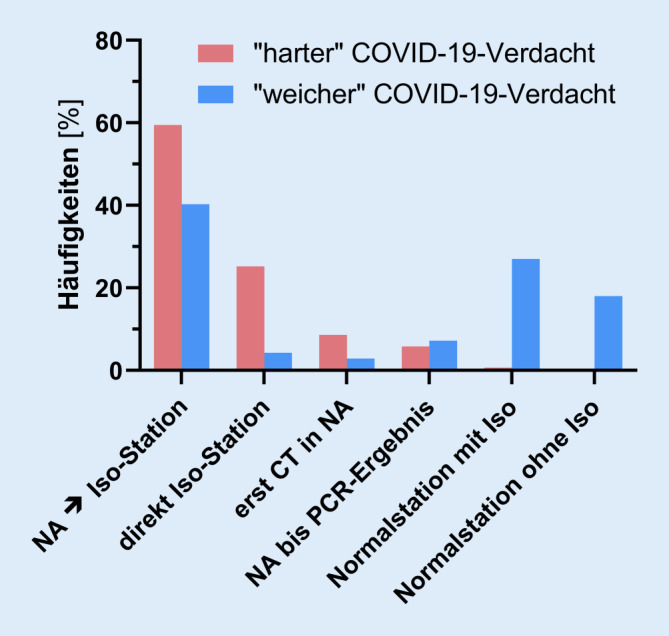

Bei der Allokation von Notfallpatienten mit Indikation zur stationären Aufnahme wurden die Teilnehmer nach dem jeweiligen Procedere bei Patienten mit hohem bzw. niedrigem klinischem Verdacht auf COVID-19 befragt (Abb. 3).

Bei Patienten mit hohem klinischem Verdacht (mutmaßliche COVID-19-Fälle) erfolgt in 59,7 % der teilnehmenden Notaufnahmen (*n* = 83) die initiale Aufnahme in den Räumlichkeiten der Notaufnahme. Anschließend erfolgt eine rasche Verlegung auf eine Isolationsstation. In 25,2 % der Fälle (*n* = 35) werden die Patienten unmittelbar auf die Isolationsstation weitergeleitet und dort aufgenommen. In 8,6 % der Notaufnahmen (*n* = 12) wird zum Zwecke der Allokation noch in der Notaufnahme eine thorakale CT-Bildgebung durchgeführt, bevor über das weitere Prozedere entschieden wird. In 5,8 % der Kliniken (*n* = 8) verbleiben die Patienten zunächst bis zum Ergebnis eines Abstrichs und zum Ausschluss relevanter Begleiterkrankungen in der Notaufnahme. In allen so praktizierenden Notaufnahmen wird ein Verbleib dieser Patienten in der Notaufnahme als sinnvoll erachtet. Eine primäre Aufnahme auf die Normalstation mit dortiger Isolation erfolgt lediglich in 0,7 % (*n* = 1) der teilnehmenden Krankenhäuser.

Wenn eine COVID-19-Erkrankung zwar klinisch unwahrscheinlich ist, aber z. B. aufgrund von respiratorischen Symptomen formal ausgeschlossen werden muss, ändert sich das Prozedere: In 40,3 % der Kliniken (*n* = 56) werden diese Patienten nach der initialen Aufnahme in der Notaufnahme ebenfalls auf eine Isolationsstation verlegt. In 27,3 % der Kliniken (*n* = 38) werden die Patienten nach Aufnahme in der Notaufnahme auf eine Normalstation verlegt und dort zunächst nicht prophylaktisch isoliert, in 18 % der Fälle (*n* = 25) erfolgt eine Verlegung auf Normalstation *mit* vorsorglicher Isolation. Ein Verbleib in der Notaufnahme bis zum Vorliegen des Abstrichergebnisses wurde von 7,2 % der Teilnehmer (*n* = 10) angegeben. In 4,3 % (*n* = 6) der Notaufnahmen erfolgt auch bei niedriger Wahrscheinlichkeit für COVID-19 sofort eine Aufnahme auf eine Isolationsstation. Nur 2,9 % der Notaufnahmen (*n* = 4) machen die weitere Allokation vom Ergebnis des Thorax-CT abhängig (Abb. 3).

### Personelle und strukturelle Aspekte

Insgesamt 97,8 % der Teilnehmer (*n* = 136) gaben an, dass der Arbeitsaufwand für Isolationspatienten seit Ausbruch der COVID-19-Pandemie zugenommen hat. Auch für Patienten ohne Verdacht auf COVID-19 berichten 60,4 % der Notaufnahmen (*n* = 84) über einen Mehraufwand.

### Sensitivitätsanalysen

**Figure 4:**
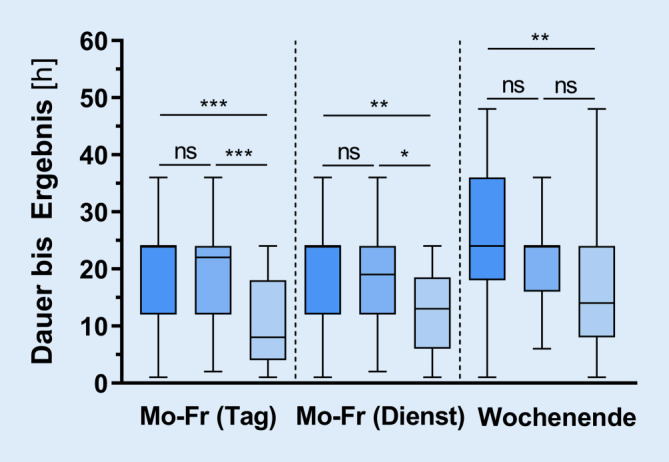

Die jeweiligen Versorgungsstufe gemäß G‑BA (Basisnotfallversorgung vs. erweiterte Notfallversorgung vs. umfassende Notfallversorgung) hatte keinen signifikanten Einfluss auf die Häufigkeitsverteilung der kategorialen/ordinalen Antworten. Ein deutlicher Unterschied zeigte sich jedoch bei der Dauer bis zum Erhalt der Abstrichergebnisse: in Notaufnahmen der umfassenden Notfallversorgung (Stufe 3) liegen die Ergebnisse signifikant früher vor als in Notaufnahmen der Stufen 1 und 2 (Abb. 4). Interessanterweise wird die ergänzende COVID-19-spezifische CT-Bildgebung in den Versorgungsstufen annähernd gleich häufig durchgeführt (Stufe 1: 34 ± 30 % vs. Stufe 2: 26 ± 25 % vs. Stufe 3: 26 ± 30 %, *p* = 0,53).

## Diskussion

Den Notaufnahmen kommt in der Pandemie eine sehr wichtige, besondere Rolle zu. In den Notaufnahmen erfolgt, neben der raschen Akutdiagnostik und -therapie, eine differenzierte Risikobewertung hinsichtlich des Risikos einer Infektion mit SARS-CoV‑2 eines jeden Patienten. Die Notaufnahmen tragen somit als „gatekeeper“ wesentlich zur Infektionsprävention innerhalb der Klinikstruktur bei. Dies geschieht im Spannungsfeld einer hohen Patientendichte bei gleichzeitig anspruchsvollen hygienischen Anforderungen und den unter Pandemiebedingungen besonderen infrastrukturellen, organisatorischen und personellen Gegebenheiten. Nicht überraschend spüren die Notaufnahmen seit Beginn der Coronapandemie einen erheblichen Mehraufwand in der täglichen Patientenversorgung. Diese Tatsache wird in der öffentlichen Diskussion um Intensivbetten nicht ausreichend gewürdigt. Die vorliegende Onlineumfrage bildet erstmalig die Notfallversorgung von COVID-19-Patienten und COVID-19-Verdachtsfällen in deutschen Notaufnahmen ab.

Die überwiegende Mehrheit der Notaufnahmen nutzt verbindliche SOP zum Thema Isolation, Diagnostik und Patientenallokation. Die eingesetzten Screeningfragebögen fokussieren sich dabei überwiegend auf die „klassische“ Symptomatik bestehend aus Fieber, Husten, Geschmacks- und/oder Geruchsverlust sowie Kontakt zu SARS-CoV-2-positiven Personen. Dieses Vorgehen deckt sich mit den klinischen Testkriterien des RKI: „akute respiratorische Symptome jeder Schwere und/oder Verlust von Geruchs‑/Geschmackssinn“ [1]. Erstaunlicherweise fragen 10 % der Notaufnahmen nicht explizit nach Dyspnoe, obwohl 48 % der frühzeitig publizierten ersten deutschen COVID-19-Fälle bei Aufnahme Dyspnoe hatten und Dyspnoe quantitativ sogar häufiger als Husten (42 %) vorlag [4].

Die prophylaktische Isolation bei gastrointestinalen (GI-)Beschwerden, insbesondere bei Diarrhö, wird nicht überall durchgeführt, obwohl 16 % der COVID-19-Patienten in der Aachener Kohorte an Diarrhö litten. Dieses Vorgehen deckt sich mit einer großen Beobachtungsstudie aus Wuhan (China), in der 10 % der COVID-19-Patienten 1–2 Tage vor der Entwicklung von Fieber und Luftnot lediglich aufgrund von Diarrhö die Notaufnahmen aufsuchten [12]. GI-Symptome und insbesondere Diarrhö sollten daher initial mit abgefragt werden, zumal Notfallpatienten mit Diarrhö ohnehin unter prophylaktischer Isolation aufgenommen werden sollten.

Inwieweit eine prophylaktische Isolation bereits aufgrund *eines* oder erst aufgrund *mehrerer* Verdachtssymptome erfolgen sollte, ist derzeit unklar. Grundsätzlich ist denkbar, dass die Prätestwahrscheinlichkeit mit der Anzahl positiver Symptome ansteigt. Italienische Autoren berichteten, dass die Trias Fieber, Husten und Luftnot während der ersten Pandemiewelle deutlich häufiger bei positiv getesteten Patienten vorlag (adjustierte Odds-Ratio: 10,02, *p* = 0,012). Diese Daten sind aber nur schwerlich auf die aktuell niedrige COVID-19-Prävalenz in Deutschland übertragbar. Aus hygienisch-präventiver Sicht ist eine sehr niederschwellige Isolation bei bereits einem Symptom bzw. Kontakt zu einer SARS-CoV-2-positiven Person vermutlich sinnvoll – erfordert jedoch erhebliche Ressourcen an Personal und Material. Dennoch präferiert auch der Großteil der befragten Notaufnahmeleiter eine niederschwellige und damit maximal sensitive Isolation bei bereits *einem* positiven Symptom in der Anamnese; sicherlich auch, um eine Transmission von COVID-19 in die Krankenhausstruktur zu verhindern.

Unabhängig davon, welche konkrete Politik die Notaufnahme- bzw. Klinikleitung in dieser Frage vertritt, sind wir der Überzeugung, dass die Entscheidung zur Isolation und SARS-CoV-2-Diagnostik keine Einzelfallentscheidung sein sollte. Die Indikationen zur Isolation müssen im Team transparent kommuniziert werden. Großer individueller Spielraum wird im Angesicht aufwändiger Isolationsmaßnahmen immer zu einer relativen Unterisolation führen. Oder anders ausgedrückt: Es besteht die Gefahr, dass auch ein motiviertes Team bei der derzeitigen Isolationsquote von geschätzt 25 % damit beginnt, bei der Indikation zur Testung und Isolation nicht vollumfänglich konsequent vorzugehen. Spätestens wenn einmal ein nosokomiales Ausbruchsgeschehen in einem Klinikum von der Öffentlichkeit hinterfragt werden sollte, ist der Verweis auf eine Einzelfallentscheidung durch Notaufnahmepersonal problematisch und kann weitreichende Folgen haben.

Zu Verhinderung von nosokomialen COVID-19-Infektionen empfiehlt das RKI für die stationäre Patientenversorgung eine Trennung in 3 „nach Möglichkeit räumlich und personell voneinander getrennte“ Bereiche: einen COVID-Bereich, einen Bereich für Verdachtsfälle sowie einen Nicht-COVID-Bereich [2]. Trotz der zunehmenden Verfügbarkeit von SARS-CoV-2-Schnelltests müssen die meisten Notaufnahmen derzeit in der Realität aber bis zum Folgetag auf die Abstrichergebnisse warten. Vermutlich ist es diesem Umstand geschuldet, dass Patienten mit „weichem“ COVID-19-Verdacht in knapp 20 % der Häuser ohne weitere Isolation auf die Normalstation verlegt werden. Obwohl wir in der Umfrage nicht explizit nach der Verfügbarkeit der vom RKI geforderten 3 Bereiche gefragt haben, lässt dieses riskante Vorgehen auf das Fehlen oder zumindest einen Engpass an 24 h-Isolationsplätzen für solche Verdachtsfälle schließen. Spätestens mit Beginn der Grippesaison wird die Vorhaltung einer ausreichend dimensionierten SARS-CoV-2-Verdachts-Unit (SVU), für die Aufrechterhaltung des Notaufnahme- und Krankenhausbetriebs essenziell werden. Auf einen erneuten „entlastenden“ Rückgang von Nicht-COVID-19-Notfällen, wie im März und April 2020 (max. −40 %), sollten wir dabei nicht spekulieren [3, 11]. Überraschenderweise werden die meisten „harten“ COVID-19-Verdachtsfälle initial in der Notaufnahme aufgenommen und erst danach auf die Isolationsstation verlegt. Dieses Vorgehen ist aus Sicht der Autoren sehr sinnvoll und unterstreicht die Kompetenz und strategische Bedeutung der Notaufnahmen für die stringente Akutdiagnostik und -therapie von u. a. COVID-19-Patienten.

Ein schwieriges Thema scheint der Umgang mit nichtanamnestizierbaren Patienten zu sein (z. B. intubierter Traumapatient, aphasischer Schlaganfallpatient, kognitiv eingeschränkter Patient). Allerdings ist eine klare Strategie hierbei unverzichtbar, da solche Patienten im Rahmen der Schockraumversorgung und CT-Diagnostik zwangsläufig mit einer Vielzahl an Mitarbeitern in Kontakt kommen und anschließend auch meist noch in systemrelevante und vulnerable Betriebseinheiten (z. B. OP, Angiographie, Intensivstation) übernommen werden müssen. Um nosokomiale Infektionen in diesen Bereichen zu vermeiden, ist eine unmittelbare, prophylaktische Isolation dieser Patienten in diesen nachgeordneten Strukturen absolut gerechtfertigt.

Gerade bei diesen Patienten kommt der Thorax-CT möglicherweise eine Schlüsselrolle zur Fortsetzung oder Beendigung der Isolationsmaßnahmen zu. Wenn man die Zahlen zu Isolation und CT-Bildgebung in dieser Umfrage hochrechnet, so führt eine Notaufnahme mit 100 Fällen pro Tag durchschnittlich etwa 8 zusätzliche CT aus COVID-19-spezifischen Gründen durch. Auch aus strahlenhygienischen Gesichtspunkten ist die CT damit offenbar kein Instrument der Primärdiagnostik, wie mancherorts noch zu Anfang der Pandemie.

**Figure 5:**
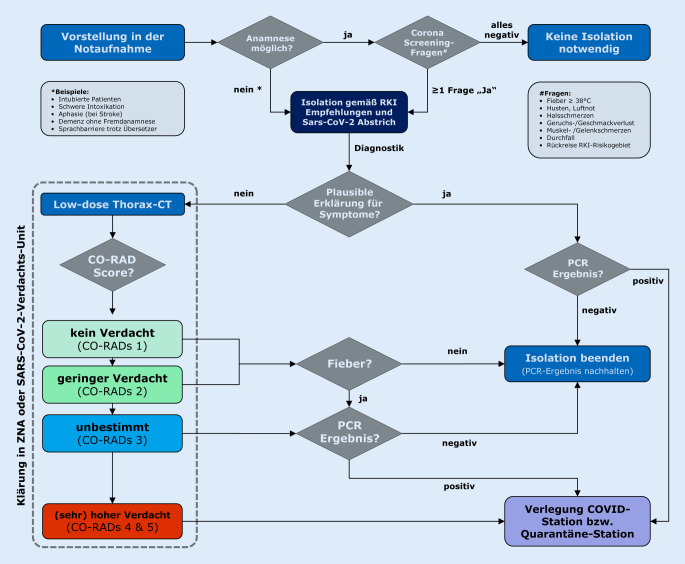

Daten des COVID-19-Bildgebungs-Register Aachen (COBRA) zeigen, dass die Niedrigdosis-CT bei Patienten mit klinischen Symptomen COVID-19 mit einer zum Abstrich vergleichbaren Sensitivität nachweisen und von anderen Erkrankungen derselben klinischen Symptomatik mit hoher Spezifität unterscheiden kann [10]. Hierbei zeigte sich, dass ein niedriger COVID-19 Reporting and Data System (CO-RADS) Score von 1 oder 2 COVID-19 in 96 % der Fälle ausschließen konnte. Der 5‑stufige CO-RADS-Score (1: sehr geringer Verdacht bis 5: sehr hoher Verdacht) wurde ursprünglich von der COVID-Arbeitsgruppe der Niederländischen Gesellschaft für Radiologie im März 2020 als standardisiertes Befunderhebungsschema zur Einschätzung einer Lungenbeteiligung bei COVID-19 vorgeschlagen. In der nachfolgenden Originalpublikation konnte ein niedriger CO-RADS-Score (1 oder 2) COVID-19 in 91 % der Fälle korrekt ausschließen. Zusätzlich wurden einige wenige Patienten mit negativem Abstrich erst durch die CT als echte COVID-19-Fälle identifiziert [6]. Ein pragmatischer Algorithmus zur Einbindung der CO-RADS-Klassifikation (inkl. nichtanamnestizierbarer Notfallpatienten) ist in Abb. 5 dargestellt. Dieser Algorithmus schließt prinzipiell auch die in der Umfrage erkennbare „Lücke“ in Bezug auf die vielerorts fehlende SOP zum Thema Entisolation.

## Limitationen

Aufgrund der hohen Rücklaufquote von > 30 % und der Beantwortung von Notaufnahmeleitern*Innen aus allen Bundesländern gehen wir durchaus von einer gewissen Repräsentativität der Daten aus. Dennoch könnte, wie bei jeder Umfrage, der Anteil thematisch interessierter Teilnehmer überwiegen, sodass die Versorgungsrealität evtl. auch unter dem hier berichteten Niveau liegen könnte.

## Fazit für die Praxis

Erfreulicherweise zeigt die aktuelle Onlineumfrage, dass die deutschen Notaufnahmen für den Moment gut aufgestellt sind, auch wenn die Isolationsschwelle an einigen Standorten möglicherweise zu hoch liegt. Spätestens in der kalten Jahreszeit sind kohärente und transparent kommunizierte Corona-SOP für alle Notaufnahmen zwingend erforderlich. Wenn nicht schon vorhanden müssen die Kliniken jetzt die Planung für SVU vorantreiben und die Skalierbarkeit von Coronastationen planen, auch wenn einige der Probleme durch die zukünftige Verfügbarkeit von Schnelltests in den Notaufnahmen relativiert werden könnten. Mit Blick auf eine mögliche Fallzahlsteigerung in den Wintermonaten wäre eine genauere Ausdifferenzierung der zu allgemein gehaltenen RKI-Empfehlungen für die spezifische Situation in der Notaufnahme wünschenswert. Der von uns vorgeschlagene Notaufnahmealgorithmus zur (Ent‑)Isolation von Verdachtsfällen könnte hierfür als Basis dienen (Abb. 5).

## Supplementary Information





## References

[1] Robert Koch Institut (2020) COVID-19-Verdacht: Maßnahmen und Testkriterien – Orientierungshilfe für Ärzte. https://www.rki.de/DE/Content/InfAZ/N/Neuartiges_Coronavirus/Massnahmen_Verdachtsfall_Infografik_Tab.html. Zugegriffen: 30. Juli 2020

[2] Robert Koch Institut (2020) Optionen zur getrennten Versorgung von COVID-19-Fällen, Verdachtsfällen und anderen Patienten im stationären Bereich. www.rki.de/DE/Content/InfAZ/N/Neuartiges_Coronavirus/Getrennte_Patientenversorg_stationaer.html. Zugegriffen: 20. Juli 2020

[3] Boender TS, Greiner F, Kocher T et al (2020) Inanspruchnahme deutscher Notaufnahmen während der COVID-19-Pandemie – der Notaufnahme-Situationsreport (SitRep). Epidemiol Bull

[4] Dreher M, Kersten A, Bickenbach J (2020). The characteristics of 50 hospitalized COVID-19 patients with and without ARDS. Dtsch Arztebl Int.

[5] Francone M, Iafrate F, Masci GM et al (2020) Chest CT score in COVID-19 patients: correlation with disease severity and short-term prognosis. Eur Radiol 30(12):6808–6817. 10.1007/s00330-020-07033-y 10.1007/s00330-020-07033-y PMC7334627 32623505

[6] Prokop M, Van Everdingen W, Van Rees Vellinga T (2020). CO-RADS: a categorical CT assessment scheme for patients suspected of having COVID-19-definition and evaluation. Radiology.

[7] Prokop M, Van Everdingen W, Van Rees Vellinga T (2020). CO-RADS—a categorical CT assessment scheme for patients with suspected COVID-19: definition and evaluation. Radiology.

[8] Ramshorn-Zimmer A, Pin M, Hartwig T (2020). Coronapandemie: Rolle der Zentralen Notaufnahme. Dtsch Arztebl.

[9] Röntgengesellschaft ATIDD (2020). Klinische Anwendung und Aussagekraft der CT bei COVID-19 Epidemie.

[10] Schulze-Hagen M, Hübel C, Meier-Schroers M (2020). Native Niedrigdosis-CT des Thorax zum Nachweis von COVID-19 – Ein systematischer prospektiver Vergleich mit der PCR-Testung. Dtsch Arztebl.

[11] Slagman A, Behringer W, Greiner F (2020). Medical emergencies during the COVID-19 pandemic. Dtsch Arztebl Int.

[12] Wang D, Hu B, Hu C et al (2020) Clinical characteristics of 138 hospitalized patients with 2019 novel Coronavirus-infected pneumonia in Wuhan, China. JAMA 323(11):1061–1069. 10.1001/jama.2020.1585 10.1001/jama.2020.1585 PMC7042881 32031570

[13] Wennmann DO, Dlugos CP, Hofschroer A (2020). Handling of COVID-19 in the emergency department : field report of the emergency ward of the university hospital munster. Med Klin Intensivmed Notfmed.

