# Supplementary material for: Isolation precautions, diagnostics and organization in German emergency departments during the 2020 COVID-19 pandemic
Source: Med Klin Intensivmed Notfmed. 2021 Jan 24;117(2):112–9. [Article in German] doi: 10.1007/s00063-021-00775-7 (PMC7829031; doi:10.1007/s00063-021-00775-7)
Supplement: Supplementary file 1 [file 63_2021_775_MOESM1_ESM.pdf]

Zusatzmaterial - Fragebogen

Isolationsmaßnahmen bei Covid-19-Patienten

1. Über wie viele Betten verfügt ihr Krankenhaus? (Zahl zwischen 10 und 3500)

2. In welche Stufe der Notfallversorgung wird Ihr Krankenhaus eingeordnet? (single choice)

   1. Basis / Stufe 1
   2. Erweitert / Stufe 2
   3. Umfassend / Stufe 3

Nur bei Antwort: Umfassend / Stufe 3

3. Arbeiten Sie an einem Universitätsklinikum? (single choice)

   1. Ja
   2. Nein

4. In welchem Bundesland sind Sie tätig? (single choice)

   1. Baden-Württemberg
   2. Bayern
   3. Berlin
   4. Brandenburg
   5. Bremen
   6. Hamburg
   7. Hessen
   8. Mecklenburg-Vorpommern
   9. Niedersachsen
   10. Nordrhein-Westfalen
   11. Rheinland-Pfalz
   12. Saarland
   13. Sachsen
   14. Sachsen-Anhalt
   15. Schleswig-Holstein
   16. Thüringen

5. Werden alle kommunikationsfähigen Patienten in der Zentralen Notaufnahme (ZNA) mittels Fragebogen oder standardisiert-strukturierter Anamnese auf Covid-19-Symptome gescreent? (single choice)

1. Ja
2. Nein

Nur bei Antwort: Ja

6. Welche Symptome werden dabei abgefragt? (multiple choice)

1. Fieber
2. Halsschmerzen
3. Gliederschmerzen
4. Schnupfen
5. Schluckstörungen
6. Husten jeder Art
7. Dyspnoe seit <14 Tagen
8. Geschmacks- und/oder Geruchsverlust
9. Abgeschlagenheit/Leistungsverlust
10. Magen-Darm-Symptome
11. Kontakt zu SARS-CoV-2 positiven Menschen
12. Aufenthalt in einem Corona-Hotspot
13. Andere (Freitext-Möglichkeit)

7. Werden die Vitalfunktionen im Rahmen des COVID-Screenings in Ihrer Zentralen Notaufnahme erhoben? (single choice)

1. Ja
2. Nein

Nur bei Antwort: Ja (Frage 7) und Ja (Frage 5)

8. Welche Vitalparameter werden erhoben? (multiple choice)

1. Herzfrequenz
2. Blutdruck
3. Sauerstoffsättigung
4. Atemfrequenz
5. Temperatur
6. Vigilanz
7. Andere (Freitext-Möglichkeit)

9. Werden alle Patienten bei positiv gescreenten Symptomen prophylaktisch isoliert? (single choice)

1. Ja, sobald mindestens ein Symptom vorliegt

2. Ja, sobald mindestens zwei Symptome vorliegen
3. Ja, sobald mindestens drei oder mehr Symptome vorliegen
4. Nein, dies ist trotz des Screenings immer eine Einzelfallentscheidung
5. Nein, reine Covid-Verdachtsfälle werden nicht vorausschauend isoliert

Nur, wenn Frage 4 mit Nein beantwortet wurde

10. Welche Symptome oder Situationen würden zu einer prophylaktischen Isolation in Ihrer ZNA führen? (Freitext)

11. Gibt es in Ihrer ZNA generell schriftlich fixierte, bindende Verfahrensanweisungen für die Behandlung von Covid-19-Patienten oder -Verdachtsfällen? (single choice)

1. Ja
2. Nein

Nur bei Antwort: Ja

12. Welche Thematiken werden in den Verfahrensanweisungen behandelt? (multiple choice)

1. Isolation
2. Diagnostik
3. Behandlung
4. Allokation / Verlegung
5. Entisolation

13. Wird bei Patienten, welche explizit nur zur ambulanten Testung auf SARS-CoV-2 kommen, diese innerhalb oder außerhalb der ZNA durchgeführt? (single choice)

1. innerhalb der Räumlichkeiten der Notaufnahme
2. außerhalb der Räumlichkeiten der Notaufnahme (bspw. Container, Testzentrum)
3. außerhalb des Krankenhauses in einem Abstrichzentrum der Stadt

14. Welche Patienten werden **innerhalb** der ZNA auf SARS-CoV-2 abgestrichen? (multiple choice)

*Beispiel für "alternative Erklärungen": Patient mit neu aufgetretenem Vorhofflimmern und Dyspnoe, kein Husten, kein Fieber

1. alle Patienten, die in die Notaufnahme kommen
2. alle Patienten, die stationär aufgenommen werden
3. alle Patienten mit positiv erfragten Symptomen, unabhängig von alternativen Erklärungen*
4. Patienten mit positiv erfragten Symptomen nur dann, wenn sie aufgenommen werden sollen

5. Patienten mit Symptomen, die eine SARS-CoV-2-Infektion klinisch hochgradig wahrscheinlich erscheinen lassen
6. Patienten erst, nachdem alternative Diagnosen ausgeschlossen wurden

15. Wer führt die Abstriche in Ihrer Notaufnahme im überwiegenden Teil der Fälle durch?

1. MFAs
2. Pflegende
3. Ärztinnen und Ärzte
4. Andere (Freitext-Möglichkeit)

16. Generell gefragt: Ist Ihr Arbeitsaufwand für Patienten mit Infektionen gestiegen? (single choice)

1. Ja
2. Nein

17. Ist Ihr Arbeitsaufwand für Patienten gestiegen, bei denen kein Verdacht auf eine SARS-CoV-2-Infektion vorliegt? (single choice)

1. Ja
2. Nein

18. Was passiert mit stabilen Patienten, bei denen eine SARS-CoV-2-Infektion klinisch **hochwahrscheinlich** ist und die stationär aufgenommen werden sollen? (single choice)

1. Verbleib in der Notaufnahme, bis das Ergebnis eines Abstrichs vorliegt
2. Aufnahme in der Notaufnahme, dann rasche Verlegung auf eine Isolationsstation bis das Ergebnis eines Abstrichs vorliegt
3. unmittelbare Weiterleitung zur Aufnahme auf eine Isolationsstation; weiteres Vorgehen von dort
4. unmittelbare Aufnahme auf eine Normalstation und dortige Isolation
5. noch innerhalb der ZNA ergänzende Risikostratifizierung mittels Throrax-CT, das weitere Vorgehen richtet sich hiernach

nur bei Antwort: Verbleib in der Notaufnahme

19. Halten Sie dieses Vorgehen für sinnvoll? (single choice)
    1. Ja
    2. Nein

20. Was passiert mit stabilen Patienten, bei denen eine SARS-COV-2-Infektion klinisch eher unwahrscheinlich ist und die aufgenommen werden sollen? (single choice)

1. sie verbleiben bis zum Ergebnis des Abstrichs in der Notaufnahme
2. Aufnahme in der Notaufnahme, dann rasche prophylaktische Verlegung auf eine Isolationsstation bis das Ergebnis eines Abstrichs vorliegt
3. unmittelbare Weiterleitung zur Aufnahme auf eine Isolationsstation; weiteres Vorgehen von dort
4. sie werden auf Normalstation verlegt und dort prophylaktisch isoliert
5. sie werden auf Normalstation verlegt und dort zunächst nicht isoliert
6. es erfolgt regelhaft eine ergänzende Risikostratifizierung mittels Thorax-CT, das weitere Vorgehen richtet sich hiernach

21. Was schätzen Sie, wieviel Prozent der Patienten in Ihrer ZNA werden aktuell (diese Woche) prophylaktisch isoliert und abgestrichen? (Zahl zwischen 0 – 100)

22. Gibt es in Ihrer ZNA einen SARS-CoV-2-Schnelltest, also eine Point-of-Care-Labordiagnostik?

1. Ja
2. Nein

Nur bei Antwort: Nein

23. Wie lange dauert es bei Ihnen **werktags zu Regelarbeitszeiten** durchschnittlich, bis die Ergebnisse eines SARS-CoV-2-Abstrichs vorliegen? (Stunden, Zahl zwischen 1 und 96)

24. Wie lange dauert es bei Ihnen werktags zu Dienstzeiten durchschnittlich, bis die Ergebnisse eines SARS-CoV-2-Abstrichs vorliegen? (Stunden, Zahl zwischen 1 und 96)

25. Wie lange dauert es bei Ihnen **am Wochenende** durchschnittlich, bis die Ergebnisse eines SARS-CoV-2-Abstrichs vorliegen? (Stunden, Zahl zwischen 1 und 96)

26. Gibt es Konstellationen, in denen in der ZNA eine zweite PCR-Testung zur Entisolation zwingend vorgesehen ist? (single choice)

1. Ja
2. Nein

Nur bei Antwort: Ja

27. Welche Konstellationen machen in Ihrer ZNA eine zweite PCR-Testung erforderlich? (Freitext)

28. Welcher Mindestabstand muss zwischen den beiden Testungen liegen? (Freitext)

29. Welche der unten stehenden Aussagen zu organisatorischen oder strukturellen Umständen treffen für Ihre ZNA zu? (multiple choice)

1. Es existiert eine räumliche Trennung zwischen einem Bereich für infektiöse, und einem Bereich für nicht infektiöse Patienten
2. Es existiert ein räumlich getrennter Bereich explizit für Covid-Patienten oder -Verdachtsfalle
3. Es gibt gesonderte Teams zur Behandlung der Patienten in infektiösen bzw. nicht infektiösen Bereichen ("Covid-Teams")

30. Haben Sie zusätzliches Personal zur Behandlung von Covid-19-Patienten erhalten? (single choice)

1. Ja
2. Nein

31. Wird die Computertomographie in der ZNA zur Covid-19-(Ausschluss-)Diagnostik eingesetzt? (single choice)

1. Ja
2. Nein

Nur bei Antwort: Ja

32. Schätzen Sie: In wieviel Prozent der Fälle mit durchgeführtem SARS-CoV-2-Rachenabstrich kommt das CT zum Einsatz? (Prozent, Zahl zwischen 0 und 100)

33. Gibt es in Ihrem Krankenhaus eine standardisierte Befundung der Thorax-CTs? (single choice)

1. Ja
2. Nein

Nur bei Antwort: Ja

34. Nach welchem Score/nach welcher Klassifikation erfolgt die Befundung? (Freitext)

35. Wird die Lungensonographie zur Covid-19-(Ausschluss-)Diagnostik eingesetzt? (single choice)

1. Ja
2. Nein

36. Gibt es festgelegte abweichende Prozesse bei nicht suffizient zu anamnestizierenden Patienten? (single choice)

Beispiele: intubierter Patient, intoxikierter Patient, Sprachbarriere

1. Ja
2. Nein
3.

37. Werden Patienten, die früher im Bypass-Verfahren die Zentrale Notaufnahme umgangen haben (z.B. STEMI, Reanimation) nun innerhalb der Notaufnahme im drive-by-Verfahren abgestrichen? (single choice)

1. Ja
2. Nein

38. Welche Materialien benutzen Sie bei der prophylaktischen Isolation von Covid-19-Verdachtsfällen? (multiple choice)

1. Chirurgische Masken
2. FFP2-Masken
3. FFP3-Masken
4. Gesichtsschutz / Face-Shield
5. Schutzbrillen
6. Handschuhe
7. reguläre Schutzkittel
8. "chirurgische" Schutzkittel

39. Wird Ihr Notaufnahme-Personal im Sinne einer Personalsurveillance regelmäßig abgestrichen? (single choice)

1. Ja
2. Nein

Nur bei Antwort: Ja

40. In welchem Abstand erfolgt die Testung des Personals? (Freitext)

41. Gab es Personalschulungen zu Themen der Corona-Pandemie? (single choice)

1. Ja
2. Nein

42. Haben Sie sonst Kommentare zum Thema der Isolation bei Corona-Patienten oder -Verdachtsfällen? (Freitext)
